# Effect of Current Density Ramping on the Growth Rate and Structure of AA2024-T3

**DOI:** 10.3390/ma15093258

**Published:** 2022-05-01

**Authors:** Peter Totaro, Boris Khusid

**Affiliations:** 1 Otto H. York Department of Chemical and Materials Engineering, New Jersey Institute of Technology, University Heights, Newark, NJ 07102, USA; ptotaro@aerotechprocessing.com; 2 Aerotech Processing Solutions, 57 Wood St., Paterson, NJ 07524, USA

**Keywords:** aerospace aluminum-copper alloys, anodizing, ramping, intermetallics, current density

## Abstract

The presented study successfully demonstrated advantages of multistep anodization of AA2024—T3. Coating properties and morphology were studied in detail for five anodization processes: a conventional Base process with a constant applied current density and processes with current density applied in one (OS1 and OS2) and five (MS1 and MS2) steps at different magnitudes during the ramp period. Due to lower oxygen infusion, processes MS1 and MS2 produced a more intact coating with reduced porosity and enhanced abrasion resistance and hardness. The presented results clearly demonstrate that starting anodization at a low voltage and then slowly ramping current density will form coatings with a higher aluminum/oxygen ratio and enhanced properties over a shorter period of processing.

## 1. Introduction

The use of 2000 series aluminum alloys in aerospace applications requires surface modifications to improve the corrosion resistance and mechanical properties of the product surface [1]. Surface modifications such as anodization, friction stir processing, and plasma electrolytic oxidation are needed to improve mechanical properties such as hardness, wear and corrosion resistance, and tribological properties [2,3,4]. Anodization is one of the most utilized methods to enhance the mechanical properties of aluminum alloys [5,6,7,8].

The presence of copper, a key alloying element, is used to form precipitates in the bulk by thermal ageing treatment [9,10] to enhance the alloy mechanical strength, fracture toughness, and fatigue properties [11,12,13]. Unfortunately, a difference between electrochemical potential of the aluminum alloy matrix and intermetallics promotes galvanic reactions that render the surface highly electro active [13,14,15]. These electrochemical differences lead to uneven growth and burning during anodization due to a spatially non-uniform distribution of electric current [11,13,14,15,16,17,18,19]. This condition is exacerbated if higher voltages and current densities are applied [11,13,14,15,16,17,18,19]. The presence of copper enhances local parasitic reactions that reduce faradaic efficiency of anodization and produce a flawed porous oxide layer [13,20,21,22,23].

The research on anodization of aluminum–copper alloys generally focuses on evaluating the influence of complex electrolyte baths, where addition of different acids such as malonic [5], carboxylic [24] fluorozirconic [25], citric [26], boric [27], and adipic [28] acids, are mixed with sulfuric acid to enhance the mechanical properties, morphology and reduce processing issues [24,25,26,27,28,29,30]. Alternatively, industrial Types II and III anodization processes of aluminum alloys require the use of sulfuric acid bath, and the application of electrolyte blends is limited [1,31,32]. Temperature and applied current/voltage are the basic process variables which can be manipulated in industry to improve the anodic coating [5]. However, variation of temperature is limited to the process of Type III, hard anodization. Therefore, the influence of applied current/voltage on the morphology and properties of anodic coatings are a subject of major interest for the industrial Type II anodizing process [1,31,32].

Anodizing current/potential influences the coating structure and properties as porosity and pore size are directly proportional to their magnitudes [33,34,35]. Anodization at high fixed voltages promote large current densities and high local heating at the beginning of the process that generates burning and reduces the film growth. This renders the coating properties unsuitable for a wide range of applications due to the presence of soft, porous coating, and conical asperities [13,21,27]. The concept of ramping in anodization is to gradually raise the current density at the beginning of the process to prevent overpotential spikes. It has been recognized that changing the current density during the process has positive effects by increasing the heat dissipation and deoxygenation at the surface of the forming anodic coating [13,19,36,37,38,39,40]. Pulse anodization, an example of varying current density, [13] was observed to improve coating properties of AA 2024-T3, but the proposed process requires expensive equipment that is not readily available in an industrial setting.

Our previous work [15] revealed the advantages of multistep anodization of AA7075—T6, (5.6–6.1% Zn, 2.1–2.5% Mg, 1.2–1.6% Cu), in a standard Type II sulfuric acid electrolyte. The composition of AA2024—T3, (4.3–4.5% Cu, 0.5–0.6% Mn, 1.3–1.5% Mg), is much different, and the difference in composition changes electrochemical behavior. It is well established that a difference in the size and mobility between copper and zinc ions changes their diffusion patterns and alters the formation of an anodic coating on these alloys [14,16,36,41]. Zinc ions accumulate at the anodic oxide/aluminum interface, while copper ions are found throughout the anodic coating and on the specimen surface [16,36,41]. The presence of zinc and copper are known to create defects in the anodic coating that are detrimental for the coating morphology and properties. Since AA7075—T6 and AA2024—T3 require different anodizing processes [14,22,31,42,43,44,45], it is difficult to predict whether advantages of stepwise anodization observed for AA7075—T6 would also benefit AA2024—T3.

The present paper aims to study the influence of applying current density in multiple steps during the ramping stage on the morphology and performance properties of an anodic coating formed on AA2024-T3 in a sulfuric acid bath. The results presented in this paper on the microstructure, growth rate, and service performance of a coating formed on AA2024—T3 show that raising the current density in five steps improves the overall process in terms of consistency, performance, and efficiency compared to a constant current anodization. We expect that a better understanding of the impact of multistep anodization on the coating properties of AA2024—T3 taken together with data on AA7075—T6 [15] would lay out the framework for the development of more efficient anodization processes on aluminum alloys.

## 2. Materials and Methods

### 2.1. Anodizing Process

**Figure 1:**
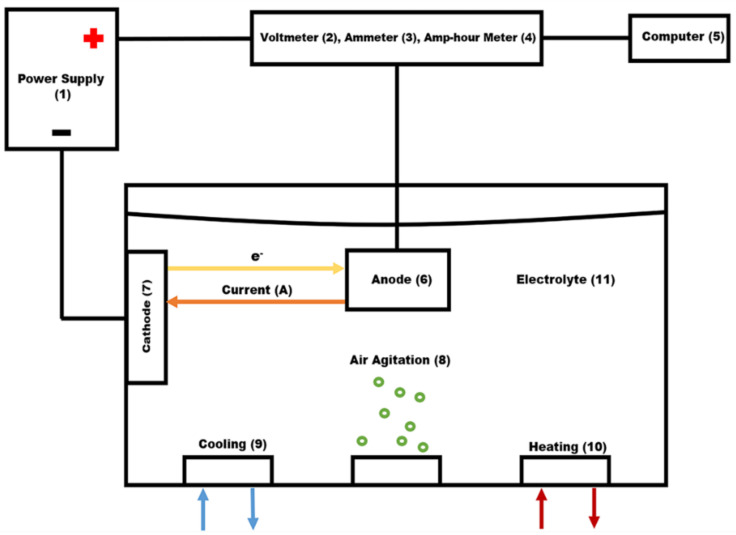
Anodizing system: (1) Power supply, (2) Voltmeter, (3) Ammeter, (4) Amp-Hour meter, (5) Computer, (6) Anode, (7) Cathode, (8) Air agitation, (9) Cooling system, (10) Heating system, (11) Electrolytic solution.

Anodization was performed in an electrochemical cell, Figure 1, in which voltage, amperage, and transferred charge are controlled with the module UPC 5000 RC-2 D P18/1000-24VN-C0, American Plating Power LLC, Florida. The UPC 5000 module was connected by a 50-mA shunt to the DC rectifier (American Plating Power LLC, Fort Myers, FL, USA) (1). The electrochemical cell consists of a polyvinylchloride tank, where the electrolyte temperature was controlled externally by a two-output/input controller.

Air agitation was performed utilizing constant low-pressure air (8). The cathodes (7) and the rack that suspended the anode (6) into the electrolyte were made of 6063 aluminum extruded bars. The cathode/anode area was kept at a 1:1 ratio measuring a total area of 0.097 m^2^. The area of each load is 0.097 m^2^ with 0.0485 m^2^ being the rack area and 0.0485 m^2^ being the part area. One cathode was mounted on each side of the tank for even distribution of an electric current.

Experiments were carried out on specimens of AA2024—T3 provided by Anacon 1st Choice, 425 W LA Cadena Dr., Riverside, CA, USA. The bulk chemical composition of this alloy given by the manufacturer is reported in the first row of Table 1. Scanning electron microscopy (SEM, JEOL JSM-7900F FE-SEM, Peabody, MA, USA) images the presence of particles on the specimen surface. The energy dispersive X-ray spectroscopy, (EDS), (AZTEC, Oxford Instruments, Abingdon, UK) measurements taken from the specimen surface in locations with particles and without particles are also reported in Table 1. They demonstrate a substantial difference between the local surface and bulk compositions in aluminum-copper alloys formed by micro-segregation and macro-segregation of alloying elements [1].

All specimens were purchased as 10 cm × 10 cm × 0.16 cm squares with a hole of 0.64 cm diameter in the middle. Specimens were then further cut to 2.54 cm × 2.54 cm × 0.16 cm squares using a diamond blade and a portable bandsaw and then deburred to reduce sharp edges. Prior to chemical processing, all specimens, anodized and non-anodized, were cleaned with reagent-grade acetone before racking to remove glue, ink, and other surface impurities. Specimens were chemically processed using an alkaline cleaner, NaOH, for 10 min, followed by a pickling process in a ferric sulfate/nitric acid bath for 5 min. Specimens were rinsed by deionized (DI) water in between each chemical step. Untreated specimens went directly to the drying step after the cleaning process. After the preparation steps, specimens were anodized at room temperature, 20 °C, for 30 min. The electrolyte (11) consisted of sulfuric acid, 180 g/L. After anodization, the specimens were cleaned in DI water and air dried.

Five anodization processes listed in Table 2 as Base, OS1, OS2, MS1, and MS2 were studied. “Base” is a conventional process with a constant electric current density; letters “OS” and “MS” mean, respectively, one-step and five-step ramping of an applied current density. Numbers “1” and “2” indicate, respectively, low, and medium current densities in ramping steps. Electric current densities applied in these processes, Table 2, span a typical range of current densities utilized in industry.

Stepwise processes were designed to explore the influence of varying the number of steps and current density during the ramp on the coating morphology and properties. In the stepwise processes, (OS1, OS2, MS1, and MS2), ramping of an applied current density was conducted for 10 min and the remaining 20-min period was carried out at a constant current (Table 2). The Base process, considered as the baseline for comparison, did not utilize a ramp. Anodic samples for testing were taken during three stages of the process. The samples were taken at 2-min intervals up to and including 10 min, and at the end of the process, 30 min. The Base process was included in the testing to study the effects of constant high current density in the same 10-min period.

The anodization process was designed to have the same electric charge passing through the system during the final stage of processing (20 min) [15]. This task was implemented to show the impact of stepwise anodization during the ramp phase of processes listed in Table 2. An electric current was calculated with the use of Equation (1) and the expected values of electric charge for the designed processes of anodization were computed with the use of Equation (2)

A = X·J,(1)

where A is the current in amperes, X is the area in m^2^, and J is the current density in A/m^2^.

C = 60·A·M,(2)

where C is Coulomb = A·sec, A is in amperes, and M is the period in minutes during which a certain current is applied, 60 is the conversion factor from minutes to seconds. Computed values of the anodizing electric current and the expected transferred electric charge for Base, OS1, OS2, MS1, and MS2 processes are summarized in Table 2.

### 2.2. Characterization Techniques

(i). The values of amperage, transferred electric charge, and actual voltage were measured using module UPC 5000 calibrated to NIST (+/−0.1 V, 0.1 A). The presented voltage, amperage, and charge are values averaged over three repeated anodizing processes.
(ii). Measurements of the coating thickness were conducted after air drying anodized specimens at a temperature of 20.0 °C for 1 h (±5 min). The coating thickness was measured using an Eddy current meter (Positector 6000, Deflesko, NY, USA). Four thickness readings were taken for every anodized specimen and the presented result is averaged over three repeated anodizing processes.
(iii). Test panels for abrasion resistance were conditioned at 46.2% relative humidity (RH) and 23.7 °C for 48 h before testing. After conditioning, panels were weighed to the nearest ±0.0001 g with an Ohaus digital balance (PA224, Ohaus, NJ, USA). After weighing, panels were positioned on the Taber abraser and allowed to re-condition for 2 h and then tested in the same conditions. Abrasion CS-17 wheels (CS-17, N Tonawanda, NY, USA) with a 1000 g load were used to abrade the surface of the specimen. Following the American Society for Testing and Materials (ASTM) D4060 standard, the Taber abraser was placed inside the SCCH high humidity chamber to condition the testing environment. Abrasion resistance was tested on two anodized specimens per process and the presented result is averaged over three repeated anodizing processes.
(iv). Micro-hardness of anodized specimens was measured by a digital Vickers tester (Durascan 50, Struers, OH, USA) with a 10 g load for 15 s. Micro-hardness readings were tested on two anodized specimens per process and the presented result is averaged over three repeated anodizing processes.
(v). Acid dissolution tests of the 7.62 cm × 7.62 cm × 0.16 cm anodized specimens were carried out in accordance with ASTM B680—80 (2019) and ASTM B 137—95 (2014) specifications. A specimen was immersed for 15 ± 0.1 min in the stirred acid test solution maintained at a temperature of 38 ± 1 °C. Two tests were performed using the following solution: 35 ± 0.5 mL of orthophosphoric acid of 85 mass%, 20 ± 0.5 g of chromic acid anhydride (CrO3) and balanced to 1000 mL of deionized water. Mass loss in mg/dm^2^ was calculated using Equation (3) [46] in accordance with ASTM B680—80 (2019):
  (3) $$\mathrm{Mass}\;\mathrm{Loss},\;\mathrm{mg}/{\mathrm{dm}}^{2}=\frac{\left(W_{1}-W_{2}\right)}{A}$$
  Total coating dissolved was calculated using Equation (4) [46] in accordance with both ASTM B 137—95 (2014) and ASTM B680—80 (2019):
  (4) $$\mathrm{Coating}\;\mathrm{Dissolved}\;\mathrm{in}\;\mathrm{Test},\;\%=\frac{\left(W_{1}-W_{2}\right)}{\left(W_{1}-W_{3}\right)}\;\times \;100$$
  where, W_1_ is the initial weight, mg, W_2_ is the weight after testing, mg, W_3_ is the weight after all coating was removed, mg, and A, is area in dm^2^.
(vi). Salt Spray (Fog) Testing is an accelerated corrosion test used to evaluate the corrosion resistance of metals and coated metals [47]. This test method, ASTM B117, has been approved for use by agencies of the U.S. Department of Defense and is widely used in the testing of anodic coatings [30,47,48,49,50,51]. Corrosion resistance testing by Salt Spray was conducted on 10 cm × 10 cm × 0.16 cm test panels that were anodized for 10 and 30 min and sealed in Anodal MS-1 New (Reliant Aluminum Products, NC, USA) for 20 min at 90 °C. One anodized specimen was tested for each anodizing process and the presented result is averaged over three repeated anodizing processes. The system used to create the corrosive environment was a Q-Fog Cyclic Corrosion Chamber (Q-Fog/SSP600, Q-LAB, FL, USA) in which specimens were exposed to a 5 wt.% NaCl fog for 336 h, as per ASTM B117 [47]. The acceptance criterion is to form less than five corrosion pits for testing over 336 h in a salt spray.
(vii). Electrochemical impedance spectroscopy (EIS) is widely used to evaluate the corrosion resistance of an anodic coating on aluminum alloys [52,53,54,55,56,57]. EIS tests were conducted with a precision impedance analyzer (Agilent 4294A, Santa Clara, CA, USA). An experiment was carried out in a two-electrode arrangement using 1.0 cm^2^ test area as the working electrode with the reference electrode Ag/AgCl, +0.197 V vs. standard hydrogen electrode. Measurements were conducted in a 3.5 wt.% NaCl solution at room temperature over a frequency range from 40 to 60 MHz with the signal amplitude of 500 mV (rms). Tests were conducted on specimens anodized for 30 min and the presented result is averaged over three repeated anodizing processes.
(viii). Surface morphology of anodized and untreated specimens was analyzed by scanning electron microscopy (SEM, JEOL JSM-7900F FE-SEM, Peabody, MA, USA). Energy dispersive X-ray spectroscopy (EDS) scanning of the alloying elements over the surface of anodized and untreated specimens was conducted using the program AZTEC, Oxford Instruments, Abingdon, UK. Two methods of imaging were used to inspect the surface morphology. Secondary electron (SE) imaging, the default setting (LED), was used for inspection of the surface topography. Back-scattered electron (BSE) imaging, BED-C setting, was used to inspect the presence of voids and defects below the specimen surface [58]. All specimens that were anodized, were gold sputtered. Point and ID EDS measurements were analyzed using 10 kV while EDS mapping measurements were analyzed using 20 kV using a 45-min acquisition time. High-magnification SEM images at 100,000× were obtained using 10 kV under LED and the height of the specimen stage, WD, of 10.0 mm. BSE and SE large-scale SEM images at 30,000× were obtained using 10 kV under LED for SE images and BED-C for BSE images, and the height of the specimen stage, WD, of 10 mm. Large- scale SEM images at 30,000× were obtained using 10 kV under LED and the height of the specimen stage, WD, of 10.0 mm. The non-anodized specimen was observed using secondary and backscattered imaging and with EDS mapping. As can be seen in Figure 9a,k large-scale SEM images at 30,000× were obtained using 20 kV under LED and the height of the specimen stage, WD, of 10.0 mm. A 20 kV beam was used for EDS mapping since mapping with a 10 kV beam produced inaccurate data for alloying elements as it utilized 𝐿𝛼-peaks for measuring their concentrations. Since the penetration of the 20 kV beam into the specimen was about 2–5 µm, EDS mapping was carried out only on specimens anodized for 30 min of processing.
  The National Institutes of Health (NIH) open-source image processing software ImageJ [59] was used to compute the porosity, pore diameter, and the interpore distance in high- magnification SEM images (100,000×) (Figure 3). The processing of an original SEM image combined a sequence of standard ImageJ procedures: Set the image scale in pixels/μm based on the scale bar equal to 100 nm; convert image type to 8-bit grayscale; reduce noise and enhance image contrast by using histogram equalization; use flat-field-correction and subtract background to correct for uneven illumination; use a locally adaptive thresholding technique to detect the boundaries between different regions in the image; select a region of interest (ROI) to automatically compute the pore size.
(ix). Equation (5) was used to calculate the efficiency of building an anodic coating, η_ox_. The anodic efficacy can be described as the ratio of the measured coating mass to the theoretical coating mass computed from the charge transferred during anodization [39]:
  (5) $$\eta_{\mathrm{ox}}=\frac{m_{2}-m_{3}}{\eta_{\mathrm{charge}}*\;\frac{M_{\mathrm{ox}}*Q\left(t\right)}{n_{\mathrm{ox}*F}}}$$
  where $m_{2}$ and $m_{3}$ are respectively the specimen mass measured after anodization, g/dm^2^, and after removal of the anodic coating, g/dm^2^; η_charge_ is the charge efficiency; M_ox_ is the molar mass of Al_2_O_3_ equal to 102 g/mol; Q(t) the cumulative charge transferred per dm^2^; n_ox_ is the number of electrons associated with the oxide formation [60]; and F is the Faraday’s constant (96,500 C/mol). Because η_charge_ is usually close to 1.0 for small anodizing systems, this value was used in calculations.
(x). X-ray diffraction (XRD) measurements were conducted on EMPYREAN, Malvern PANalytical, UK at an incident angle 2° in parallel beam geometry with grazing technique, to reduce the signal from the substrate and enhance the signal from the coating, with Rigaku Optima IV diffractometer, Rigaku Analytical Devices, Wilmington, MA, USA, equipped with CuKα radiation. Two scans at the operating parameters of 40 mA, 45 kV and 0.5° min^−1^ scanning speed were conducted for every anodized specimen and the presented result is averaged over three repeated anodizing processes. The instrumental broadening of XRD peaks were measured using the National Institute of Standards and Technology Standard (NIST) Reference Material^®^ 1976c consisted of a sintered alumina disc [61].
(xi). The reported percentage difference between two measured values in all tests, val_1_ and val_2_ was calculated using Equation (6):
  (6) $$\mathrm{Difference}\;(\%)=100\;\times \;\frac{\mathrm{ABS}({\mathrm{Val}}_{1}-{\mathrm{Val}}_{2})}{({\mathrm{Val}}_{1}+{\mathrm{Val}}_{2})/2}$$
(xii). In the statistical analysis of data, measurements of the anodic coating characteristics were arranged in comparison groups: Group 1 (Base, OS1, OS2) and Group 2 (MS1, MS2) as well as in two groups to compare the outcome of processing for 10 min and 30 min. The F-test of equality of variances was used to determine whether both populations have the same variance [62]. The null hypothesis of an experiment states that a difference between measurements within a particular group compared with the other group appeared by chance. The alternative hypothesis is that this difference was influenced by differences in anodizing processes. The following three-step procedure performed with Analysis of Variance (ANOVA) Microsoft Excel was used for testing the null hypothesis: (1) Compute the mean and variance of measurements for each of the two groups; (2) compute the overall mean and variance for both groups taken together; and (3) compute the F factor as the ratio between the mean variability of measurements within one group and the mean variability of data within both groups taken together. The value of the F factor will be large only if the variability between the groups is large compared to the variability within both groups taken together. The number of measurements in each group, the total number of measurements, and the chosen alpha level, α, yield the confidence level $100\cdot \left(1-\alpha \right)\%$ [62]. There are two criteria for rejecting or accepting the null hypothesis. One is to calculate $F_{\alpha }$ that is a function of α: F should exceed $F_{\alpha }$ for the null hypothesis to be rejected. The other is to calculate the $P_{\alpha }$—value that is a function of α for rejecting, $\alpha >P_{\alpha }$, or accepting, $\alpha \leq P_{\alpha }$, the null hypothesis. The reported results of F-tests were conducted for α=0.05 corresponding to the confidence level of 95%.

## 3. Results and Discussion

### 3.1. Coating Performance Characteristics

#### 3.1.1. Coating Thickness

Anodic coating thickness was measured using an Eddy current meter. Table 3 reports coating thickness measurements (µm) obtained from processes Base, OS1, OS2, MS1, and MS2.

Three time periods were chosen to describe the effect of applying multi-stepped current density vs. constant current density on coating build up. The first period, the 10-min ramp, was measured in five, 2-min increments. The current densities used were different for each ramp process and are referenced in Table 2. The final stage of anodization was carried out for 20 min at the current density stage of 180 A/m^2^ for every process. The rightmost column in Table 3 presents the overall coating thickness.

Multi-stepped processes, MS1 and MS2, formed thicker coatings than coatings produced in the Base process. Noteworthy, the coating thickness formed during the final current density stage for processes, MS1 & MS2, was greater than over the entire OS1 process. The total thickness formed by Base process, 10.9 µm, processes MS1 and MS2 produced 93.6% and 89.0% of that coating thickness in 33% less time. All processes utilizing ramping outperformed the Base process in coating thickness formed during the final 20-min stage of processing. The results of statistical analysis presented in Table 3 demonstrate that a difference between the influence of anodizing processes in Group 1 and Group 2 on the coating thickness is statistically significant as $F>F_{0.05}$ and $0.05>P_{0.05}$.

#### 3.1.2. Abrasion Resistance of Anodic Coating

Abrasion resistance was characterized by calculating the weight of the anodic coating removed following 1000 abrasive cycles. Table 4 lists the weight loss measurements for each specimen.

**Figure 2:**
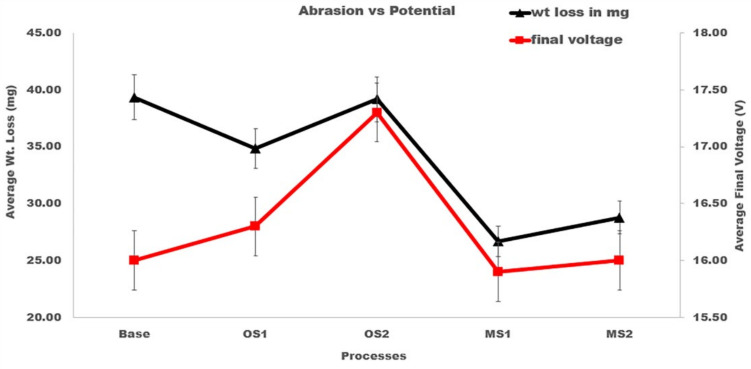
Average wt. loss, mg, versus average final voltage, V. The triangle shape represents the average wt. loss and the square, the average final voltage. A reduction in wt. loss with lower final voltage is noticed in MS1 and MS2.

The presented results demonstrate that processes utilizing lower applied voltage, OS1, MS1, and MS2 provided a higher abrasion resistance by 12.1%, 38.2%, and 31.2%, respectively, when compared to the Base process. The results of statistical analysis presented in Table 4 demonstrate that a difference between the influence of anodizing processes in Group 1 and Group 2 on the abrasion resistance is statistically significant as $F>F_{0.05}$ and $0.05>P_{0.05}$. Plots in Figure 2 demonstrate that as the final voltage decreased, the abrasion resistance increased.

**Figure 3:**
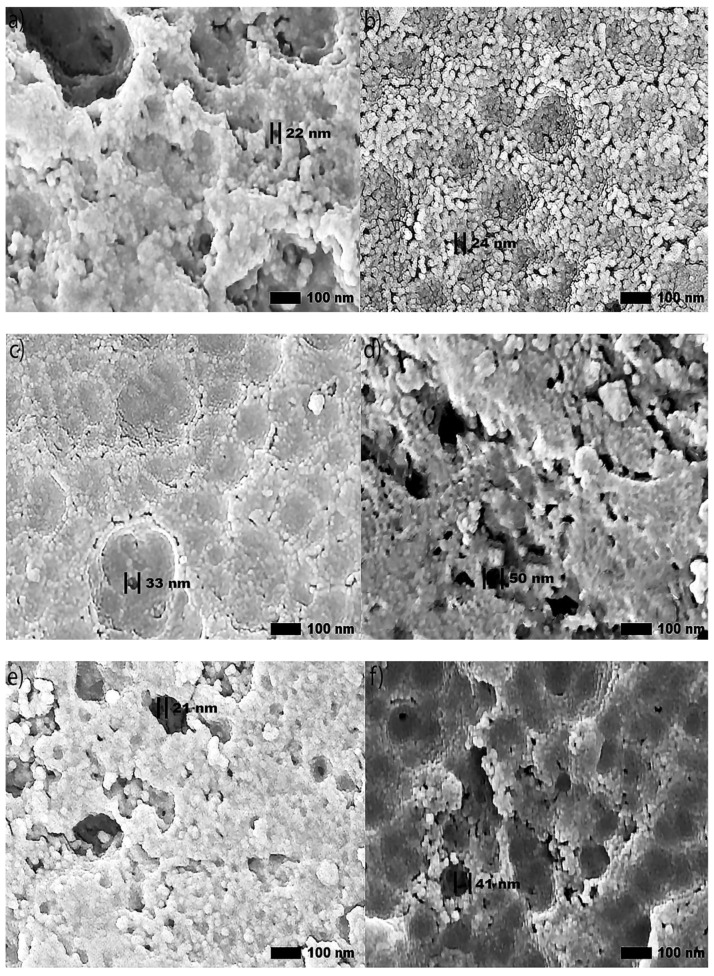

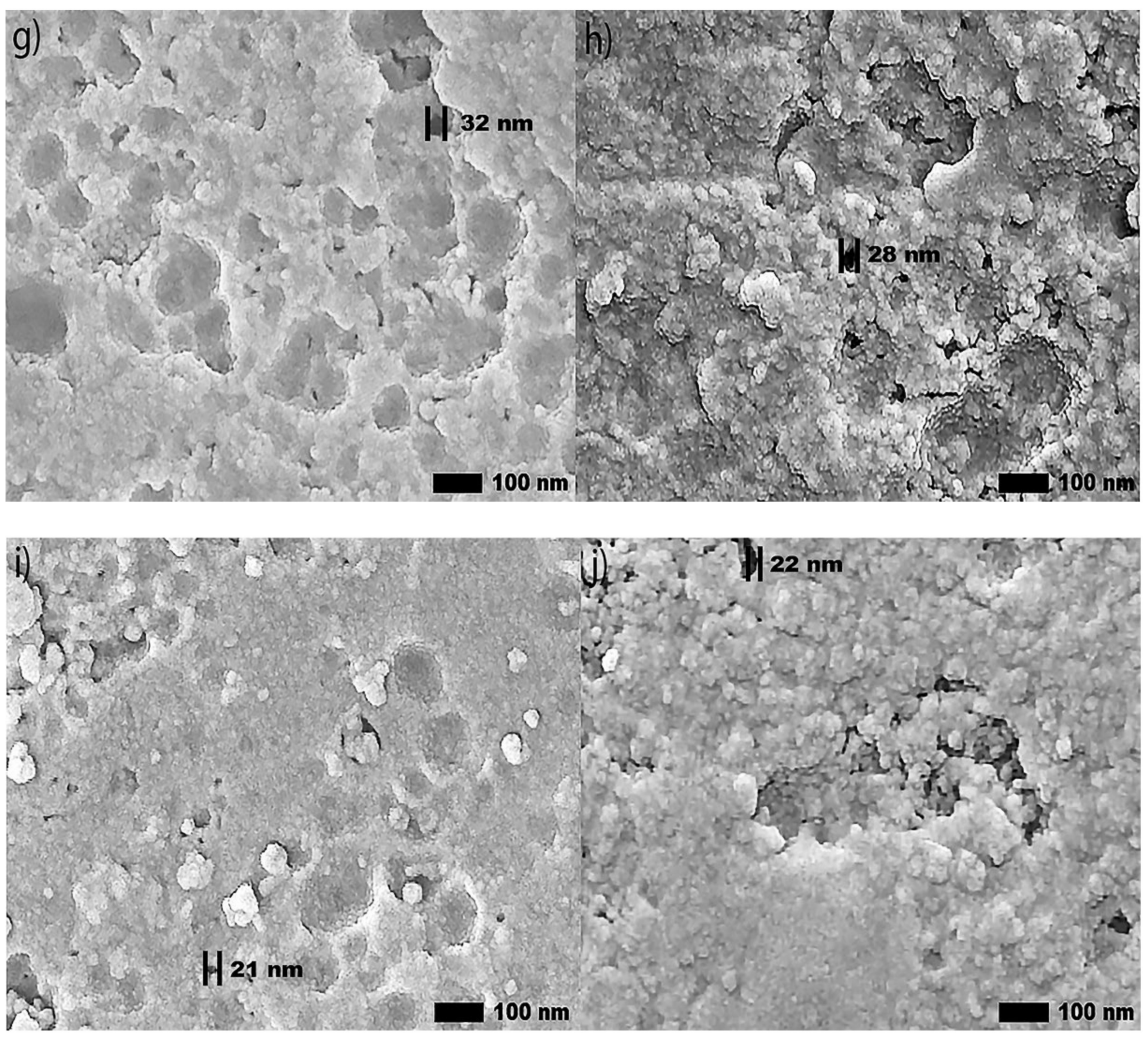
(**a**–**j**). Secondary SEM images (100,000×) of the surface morphology reveal circular pores created in the anodization process, respectively, in Base (**a**,**b**), OS1 (**c**,**d**), OS2 (**e**,**f**), MS1 (**g**,**h**), and MS2 (**i**,**j**) processes. Left images show specimens anodized for 10 min and right images show specimens anodized for 30 min. Computations were conducted with software ImageJ [59].

High-magnification SEM images (100,000×) of anodic coatings posted in Figure 3 demonstrate a correlation between the increased abrasion resistance and the improvement of the coating morphology. Specifically, these large regions of cracking and pitting formed during anodization are more prominent in the coatings produced by Base and OS2 processes. Coatings subjected to higher voltages, developed areas with surface asperities and porosity. Areas that exhibit these features will become loose upon abrasion.

#### 3.1.3. Microhardness of Anodized Specimens

Table 4 lists the average microhardness values in MPa. It is noticeable that the coating hardness increases with the coating thickness. Compared to standard process Base, the hardness of coatings formed in processes OS2, MS1, and MS2 increased by 6.6%, 8.7%, 12.6%, respectively. Process OS1 had an 11.1% reduction in hardness compared to the Base. Hardness measurements presented in Table 4 indicate that the hardness of the coating is dependent on the coating thickness. The results of statistical analysis presented in Table 4 demonstrate that a difference between the influence of anodizing processes in Group 1 and Group 2 on the microhardness is statistically significant as $F>F_{0.05}$ and $0.05>P_{0.05}$.

#### 3.1.4. Anodic Coating Resistance to Acid Dissolution

The resistance to dissolution was characterized by immersing specimens in a chromic and phosphoric acid bath for 15 min and then calculating the weight of the anodic coating removed. Measurements are reported in Table 4 in the following terms: weight loss per coating area, mg/dm^2^, loss of total coating, %, and weight loss per micron, mg/µm. Anodic coatings were completely dissolved on all specimens anodized for 10 min. Specimens anodized for 30 min in Base, OS1 and OS2 processes lost more than 99.0% of its anodic coating while specimens anodized in MS1 and MS2 processes lost 95.2% and 96.2%, respectively. As BSE imaging in Figure 5 illustrate, a reduction of porosity and surface asperities also increased resistance to acid dissolution by reducing the available interstitial regions of a coating to be dissolved. Measurements presented in Table 4 demonstrate that anodic coating resistance to acid dissolution increases with coating thickness. The results of statistical analysis presented in Table 4 demonstrate that a difference between the influence of anodizing processes in Group 1 and Group 2 on the acid dissolution of coatings is statistically significant as $F>F_{0.05}$ and $0.05>P_{0.05}$.

#### 3.1.5. Corrosion Resistance of Anodized Specimens

##### Salt Spray (Fog) Testing

Salt Spray (Fog) testing was conducted on sealed specimens anodized for 10 min and 30 min. For specimens anodized for 10 min, coatings produced by the Base, OS1 and MS2 processes formed 2 ± 1 pits per process. Coatings produced by the OS2 and MS1 processes, formed just one pit per process. For specimens anodized for 30 min, no pits formed on coatings produced in the Base, OS1, OS2, and MS1 processes, while the coating produced from the MS2 process formed an average of 2 ± 1 pits. The presented results indicate that the coating corrosion resistance is mainly attributed to the ability of sealing the pores and improved with forming a thicker anodic coating.

##### Electrochemical Impedance Spectroscopy (EIS)

**Figure 4:**
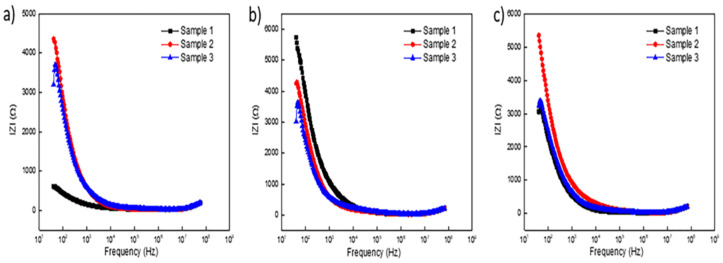

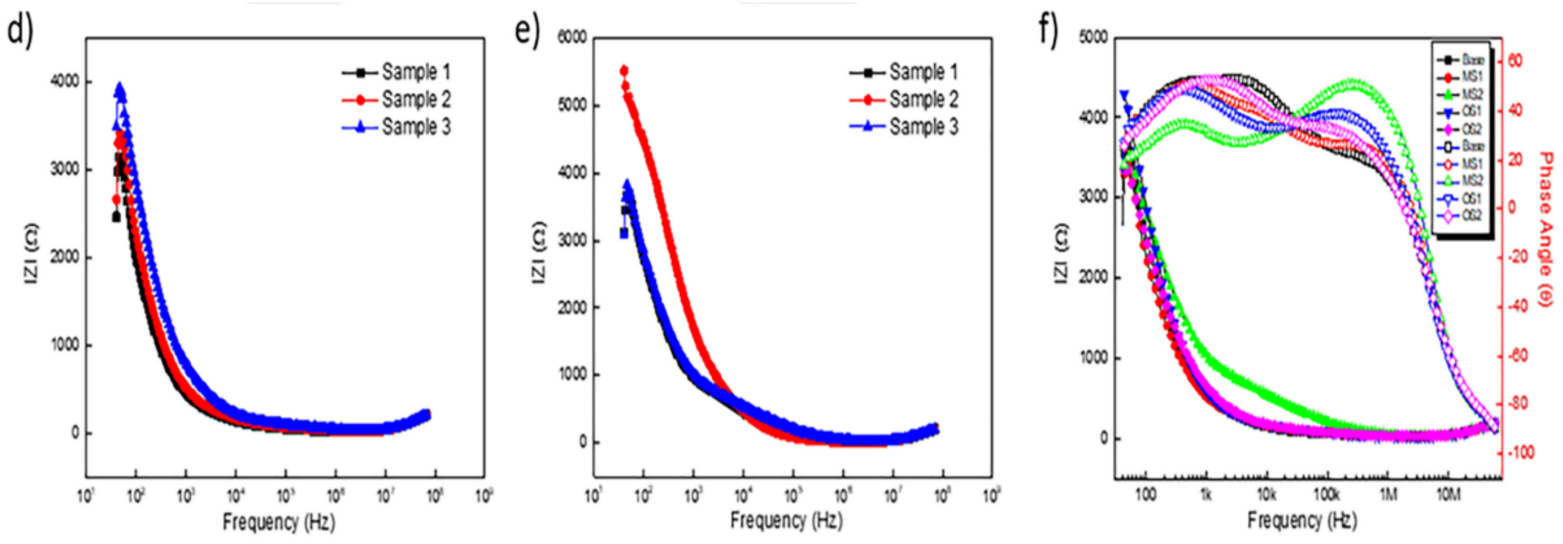
(**a**–**f**). EIS spectra obtained for unsealed specimens anodized for 30 min in processes Base (**a**), OS1 (**b**), OS2 (**c**), MS1 (**d**), MS2 (**e**), and Bode Plots (**f**).

Plots in Figure 4a–f report the EIS response of unsealed specimens anodized for 30 min. The overall EIS spectra appear to be minimally affected by the anodic coating morphology as all impedance moduli followed a similar cycloid curve with increasing frequency. In the EIS spectrum of anodic specimens, the low frequency region represents properties of the barrier layer and localized corrosion sites, while the high frequency range represents the behavior of the porous layer of the coating [63]. Plots in Figure 4c,e for coatings formed in OS2 and MS2, respectively, exhibit a slightly elevated impedance moduli in the low frequency region below 1 kHz. Bode plots in Figure 4f illustrate the EIS spectra of specimens anodized in Base, OS1, OS2, MS1, and MS2 processes. In Figure 4f, the impedance moduli decreased with increasing frequency for all processes. The phase angles rapidly depress at high frequencies, with the exception of MS2, that slightly elevates in the region 10 kHz–1 MHz and then reduces similarly to other anodizing processes.

#### 3.1.6. SEM/EDS Analysis

Secondary SEM images (100,000×) of anodic coatings posted in Figure 3 show the surface of coatings anodized in Base, OS1, OS2, MS1, and MS2 processes over 10 and 30 min. The results demonstrate that multistep ramping of applied current density substantially improved the coating morphology. In all specimens, coatings became more undulated and porous with distinct fragmentation patterns formed during the final stage of anodization. These undesired features are likely caused by increasing nonuniformity in the growth of a coating due to higher voltages used at this stage of the process. Coatings formed in the Base, OS1, and OS2 processes during 10 min exhibited a porous morphology. This condition was greatly exacerbated during the final period of the 30-min processing. The MS1 and MS2 processes produced coatings that were considerably smoother and less undulated (Figure 3). The presented results demonstrate that utilizing multistep ramping at the beginning of an anodization process promotes the formation of an anodic coating with a finer morphology and a lower porosity compared to processes without or with a single ramp. Higher potential applied at the beginning of the process inhibits anodic oxidation at certain locations, likely due to the presence of copper and other alloying elements in the coating. Copper contamination can promote uneven film growth and increased electrical resistance.

**Figure 5:**
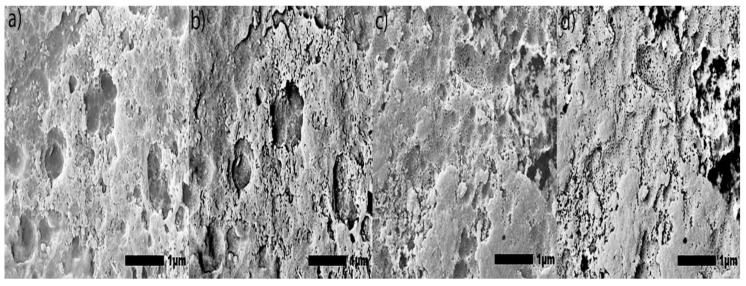

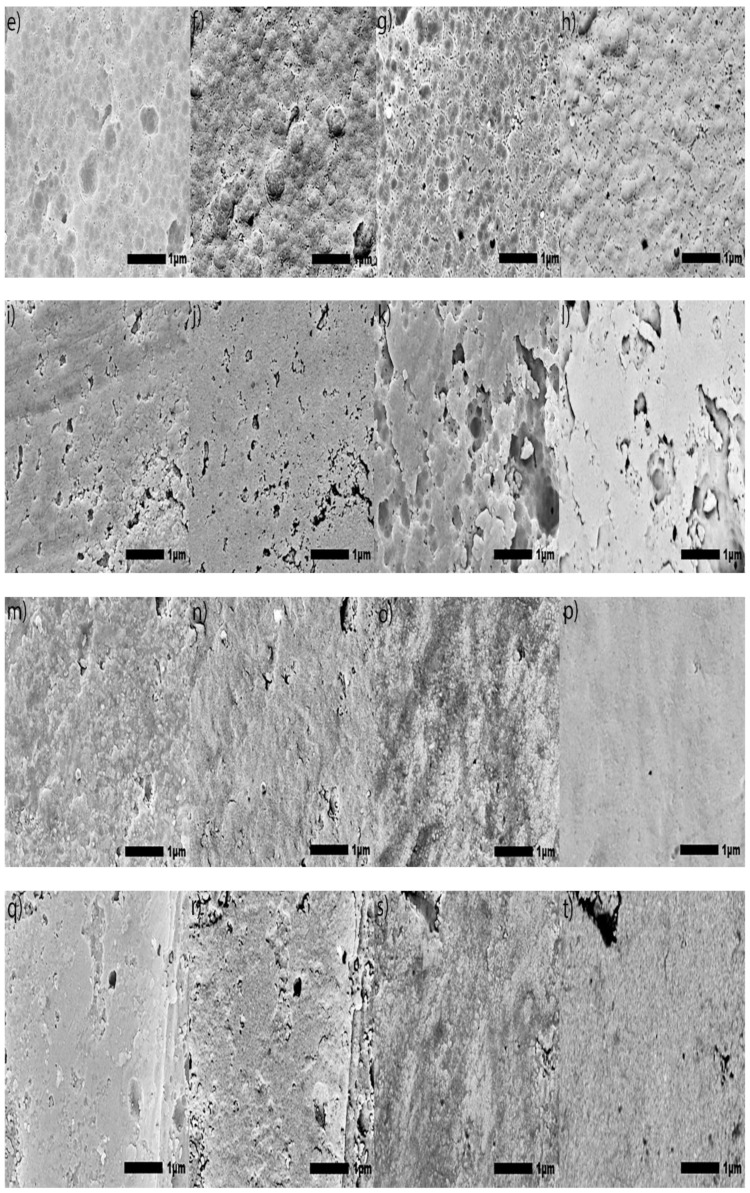
(**a**–**t**). Large-scale SEM images (30,000× magnification) of the coating surface were obtained with the use of back-scattered electrons (BSE) imaging and secondary electrons (SE) imaging: 10 kV under LED for SE images and BED-C for BSE images were used, and with a height of the specimen stage, WD, of 10 mm. Specimens anodized respectively in [Base]: (**a**) 10-min SE, (**b**) 10-min BSE, (**c**) 30-min SE, (**d**) 30-min BSE; [OS1]: (**e**) 10-min SE, (**f**) 10-min BSE, (**g**) 30-min SE, (**h**) 30-min BSE; [OS2]: (**i**) 10-min SE, (**j**) 10-min BSE, (**k**) 30-min SE, (**l**) 30-min BSE; [MS1]: (**m**) 10-min SE, (**n**) 10-min BSE, (**o**) 30-min SE, (**p**) 30-min BSE; and [MS2] (**q**) 10-min SE, (**r**) 10-min BSE, (**s**) 30-min SE, (**t**) 30-min BSE.

The SE and BSE SEM images posted in Figure 5a–t provide a large-scale view of the surface of specimens anodized in Base, OS1, OS2, MS1, and MS2 processes for 10 and 30 min.

The BSE imaging provides a contrasting aspect of the morphology to illustrate the presence of voids, defects, and porosity of the coating, while the SE imaging illustrates the specimen surface topography. Taken together, both types of imaging provide a detailed view of the coating morphology and surface anomalies. In Figure 5a–d, the coating produced by the Base process is populated with cracks, voids, and asperities across the entire surface. In Figure 5i–l coatings produced by the OS2 process exhibit similar defects, but to a lesser extent. Coatings formed in the OS1 process (Figure 5e–h) were intact after the ramping period, but eventually became more porous with a longer anodizing time. Processes MS1 and MS2 produced a coating with fewer voids and imperfections as gradual increases in the applied current density allow for the formation of more intact, void-free coatings (Figure 5m–t).

**Figure 6:**
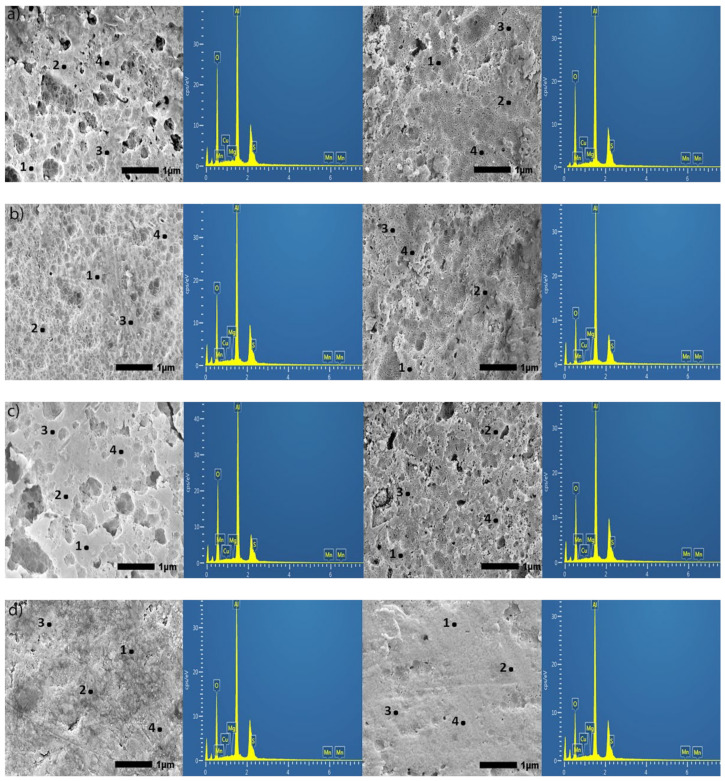

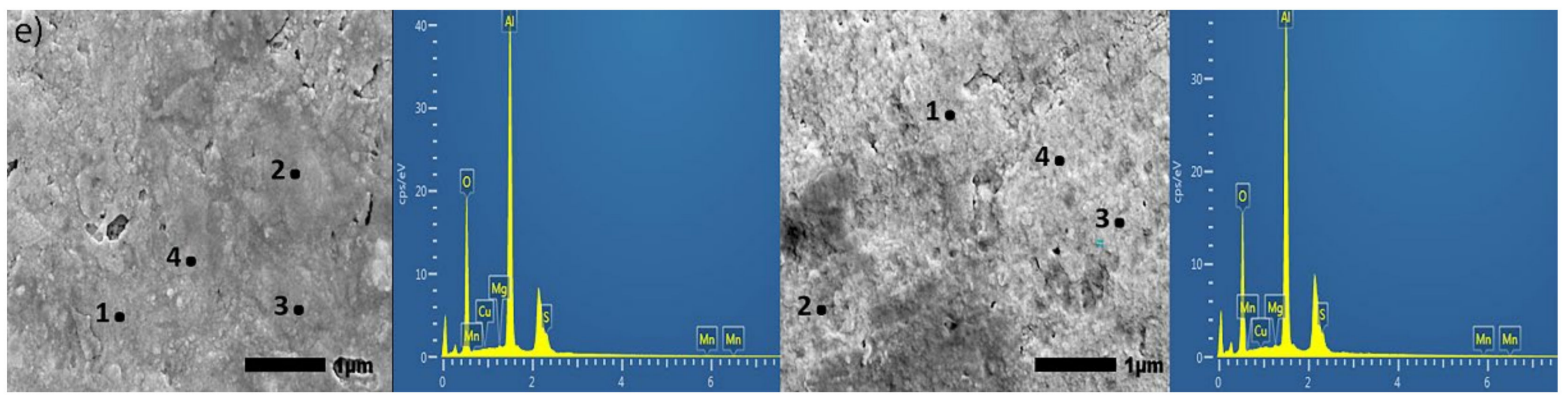
(**a**–**e**). Secondary SEM images (30,000×) of specimens anodized in Base (**a**), OS1 (**b**), OS2 (**c**), MS1 (**d**), and MS2 (**e**) processes; for 10 min, left image, and for 30 min, right image. The EDS analysis was performed at 12 sampling sites in flat, non-pitted regions of the anodic coating. Sites labeled 1–4 are representations of the 12 sites chosen. The EDS spectrum shown on the adjacent image is data for site 1.

SEM images posted in Figure 6 provide a large-scale view of the surface of specimens anodized in Base, OS1, OS2, MS1, and MS2 processes for 10 and 30 min.

The energy dispersive X-ray spectroscopy (EDS) analysis was performed at 12 sampling sites in flat, non-pitted regions. In Figure 6, sites labeled 1–4 are representations of the 12 sites chosen. Two constraints impede the quantification of EDS measurements presented. First, the heights of the peaks for elements Cu, S, Mn, Mg, etc., (Figure 6) vary from site to site as shown in these images due to the inhomogeneity of the coating. Next, the 10 kV beam was used for measurements as the depth of the 20 kV beam penetration was around 2–5 µm. It therefore requires the use of Lα characteristic X-ray (keV) for measuring concentrations of these elements that could render the error as high as 3–5 wt.% due to peak overlap [64]. For these reasons, reported concentrations of Cu, S, Mg, and Mn can be used only as reference points.

**Figure 7:**
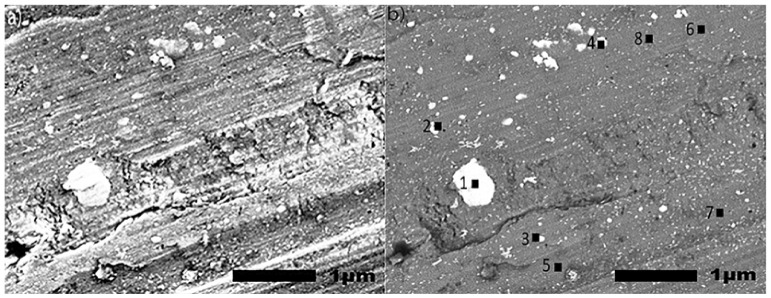
(**a**,**b**). SEM images (30,000×) utilizing secondary electron (SE) imaging (**a**) and back scattered electrons (BSE) (**b**) of a non-anodized specimen. The EDS analysis was performed at eight locations (4 with particles and 4 without particles) to evaluate the surface composition of a non-anodized specimen.

Images of an untreated specimen acquired by SE and BSE imaging are posted in Figure 7a,b, respectively. The specimen was chemically cleaned with the same method as the other specimens, to provide detail on the surface prior to anodization. EDS measurements shown in Figure 7b were taken in locations with particles (1–4) and without particles (5–8). Results presented in Table 1 show the composition of these regions. The SE image in Figure 7a and BSE image in Figure 7b demonstrate a difference in local surface compositions, as the lighter color shows particles with a lower content of aluminum and a higher content of O, Cu, Mg, Mn, and Fe compared to the surrounding aluminum rich regions (Table 1).

**Figure 8:**
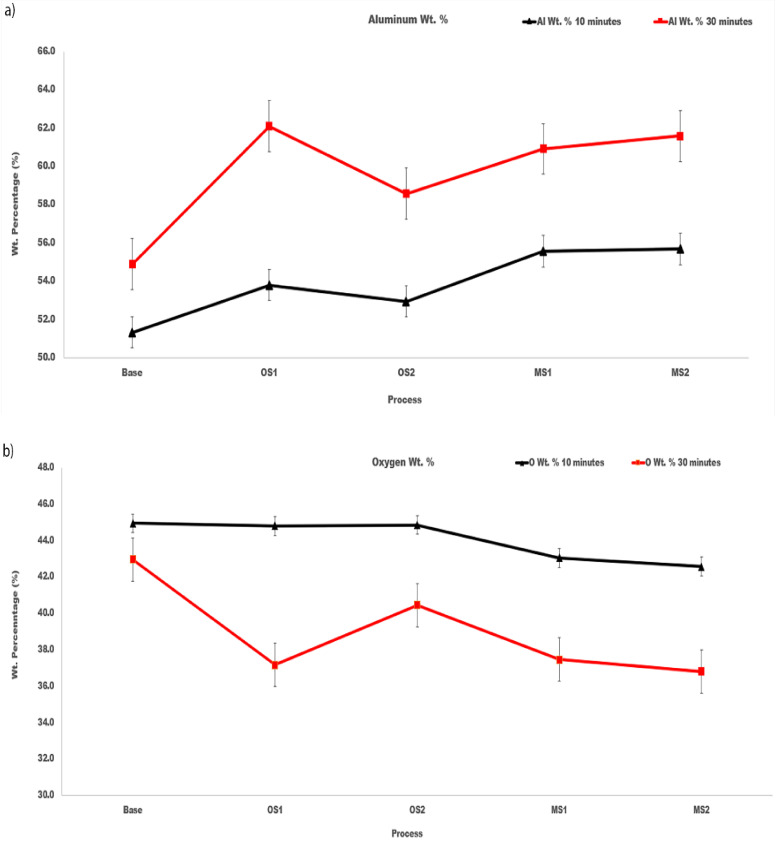
(**a**,**b**). The amount, wt.%, of (**a**) aluminum and (**b**) oxygen at the coating surface of specimens anodized in Base, OS1, OS2, MS1, and MS2 processes over 10 min and 30 min. Reported values were averaged over 12 sites depicted in Figure 6.

Plots in Figure 8 demonstrate the effect of anodizing processes Base, OS1, OS2, MS1, MS2 on the content of aluminum and oxygen in coatings formed over 10 min and 30 min.

For all processes, the coatings formed in the 30-min anodizing process had higher wt.% of aluminum and lower wt.% of oxygen when compared to the 10-min process. The atomic Al/O ratios in coatings formed by anodization for 10 min and 30 min are listed in Table 5. As can be seen in Figure 8a,b and Table 5, differences between the aluminum and oxygen contents in coatings created by different anodizing processes exceed variations of their concentrations within a coating built under the same anodizing conditions. Compared to Base process, stepwise processes MS1 and MS2 created coatings with the aluminum content larger by 4–7 wt.% and the oxygen content lower by 2–6 wt.%.

The Al/O ratio increases with longer anodizing time and all values are greater than the Al/O stoichiometric ratio of 0.67 in the Al_2_O_3_ oxide. The results of statistical analysis presented in Table 5 demonstrate that a difference between the influence of anodizing processes in Group 1 and Group 2 on the Al/O ratio is statistically significant as $F>F_{0.05}$ and $0.05>P_{0.05}$.

**Figure 9:**
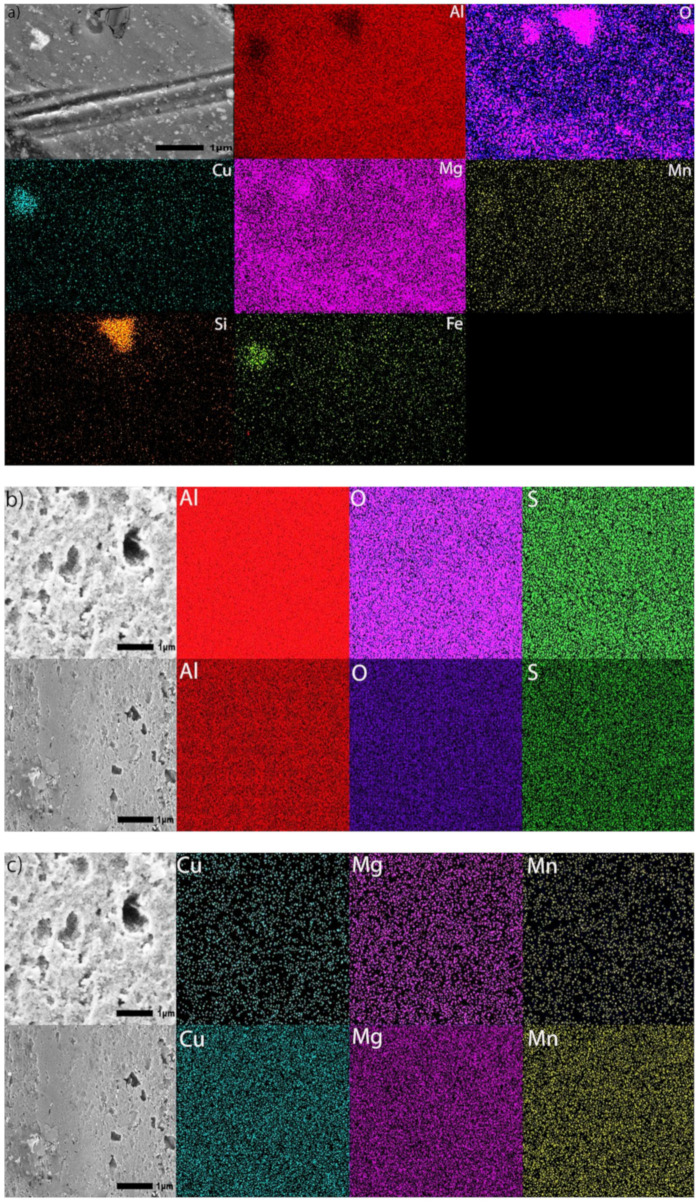

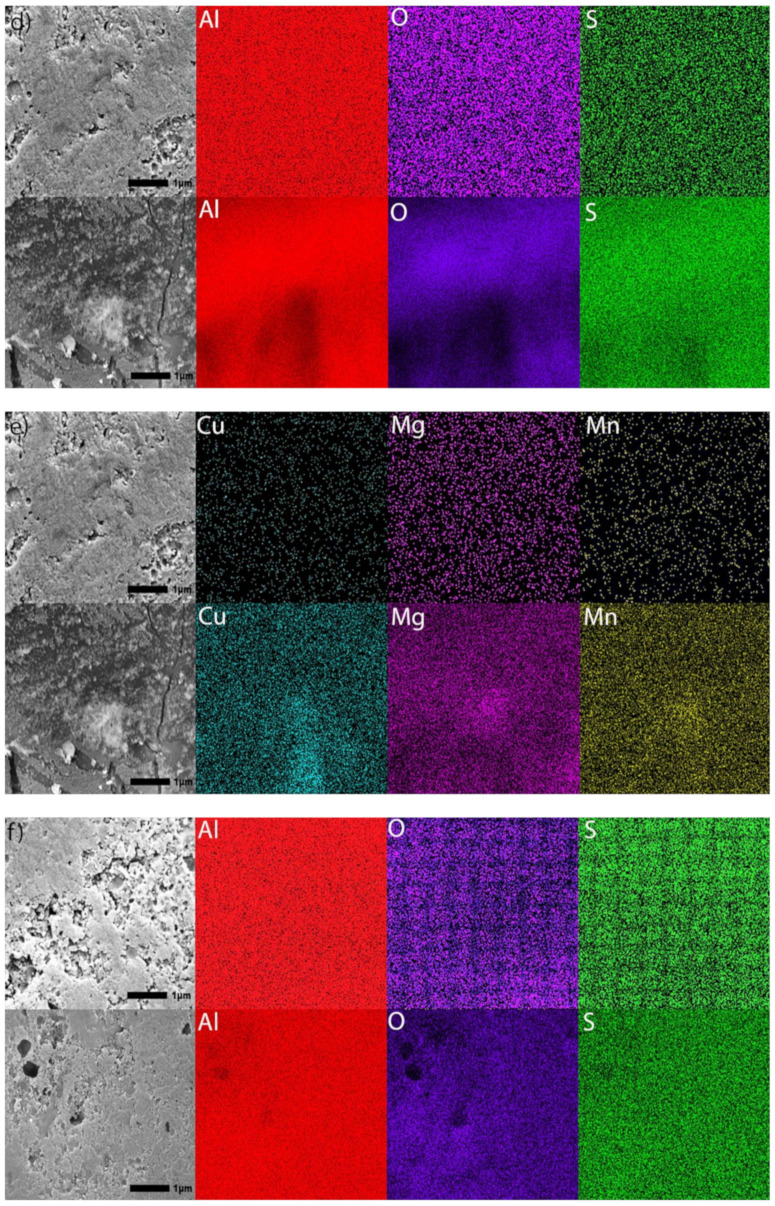

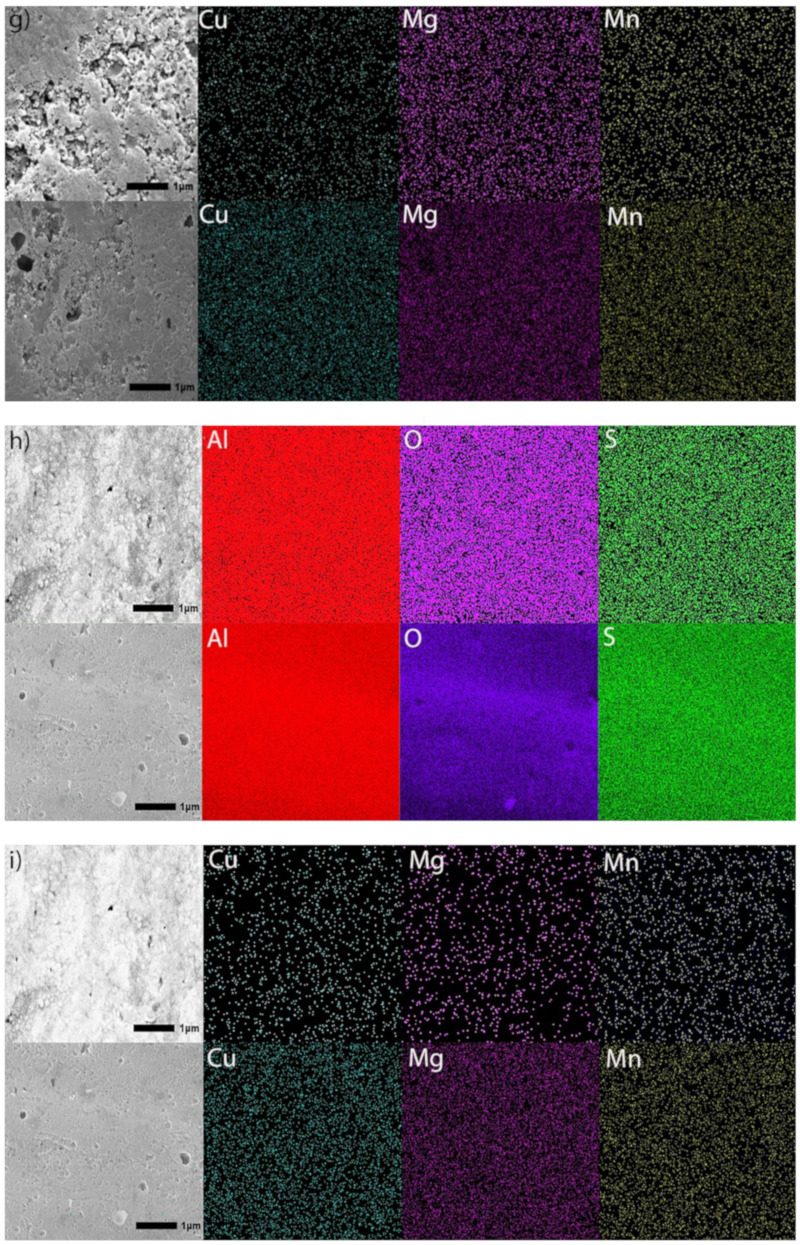

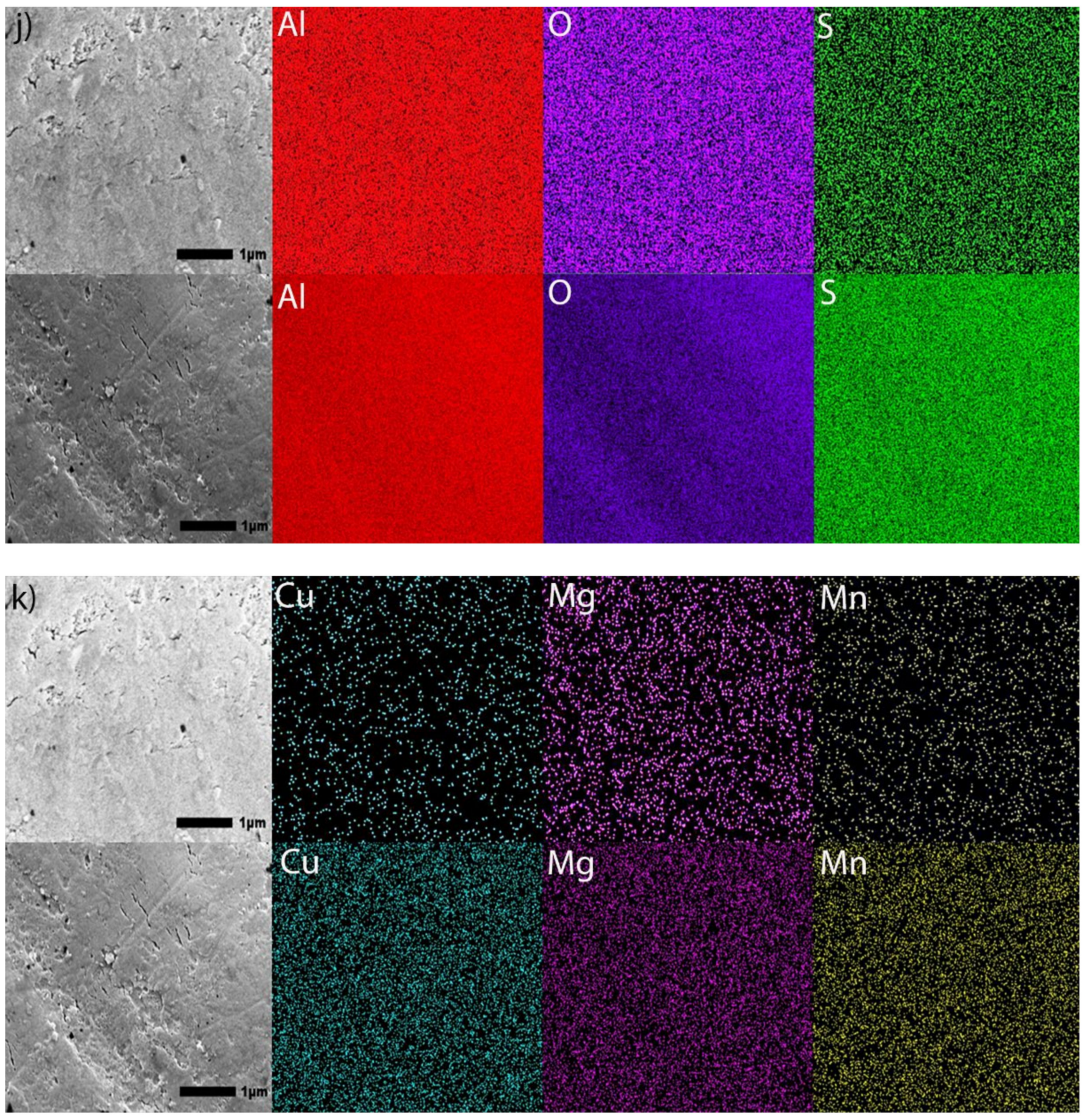
(**a**–**k**). SEM images at 30,000× and EDS mapping images of the surface of (**a**) an untreated specimen and specimens anodized for 30 min in processes Base (**b**,**c**), OS1 (**d**,**e**), OS2 (**f**,**g**), MS1 (**h**,**i**), and MS2 (**j**,**k**). Images were obtained using 20 kV under LED. The acquisition time was about 45 min to acquire 200 counts/pixel (top row) and 325 counts/pixel (bottom row). Colors representing elements are as follows: aluminum (red), oxygen (purple), copper (teal), magnesium (pink), manganese (yellow), silicon (orange), iron (lime) and sulfur (green).

SEM images in Figure 3 and Figure 5, illustrate that utilizing multistep ramping of current density at the beginning of anodization promotes the formation of coating compositions higher in aluminum and lower in oxygen (higher Al/O ratios, Table 5). The EDS maps presented in Figure 9 were acquired in two EDS runs. They illustrate the distribution of elements over the surface of specimens anodized in processes Base, OS1, OS2, MS1, and MS2 as well as over the surface of an untreated specimen. The EDS mapping image of an untreated specimen (Figure 9a) shows the presence of Fe and Si, mainly in two large particles. The dark particle has a higher concentration of Si and O and is likely a SiO_2_ particle, whereas the light particle has a higher concentration of Cu and Fe and is likely an AlCuFe particle [13]. However, Fe and Si were not observed on the surface of anodized specimens. The presence of sulfur seen in images posted in Figure 9 is likely caused by the absorption of SO_4_^2−^ ions penetrating into the anodic coating from the electrolyte. The EDS maps in Figure 9 clearly demonstrate that concentrations of alloying elements in anodic coatings are very small; however, there are slight differences in the distribution of trace elements on the surface of coatings formed by different anodizing processes.

Table 6 lists the values of the pore diameters, interpore distance, and pore density computed from SEM images (100,000×) in Figure 3 with the use of software ImageJ [59].

Pore size measurements are illustrated in Figure 3a–j. Pore density was calculated using Equation (7) [65]:

(7) $$N=\frac{2\;x\;10^{6}}{\sqrt{3}\left(D_{\mathrm{int}}{}^{2}\right)}$$

where N is the number of pores per unit area in µm^2^ and D_int_ is the interpore distance in nm. The average pore diameter increase was 49.9%, with the largest increase 64.4% found in the MS2 process. Pores formed at the beginning of anodization have little or no beveling at the pore wall to the adjacent region. The growth of a pore diameter could be attributed to a higher rate of dissolution around a pore during later stages of processing. The observed dependence of the interpore distance and pore density on the applied initial voltage is consistent with Equation (8) [66] for the porosity of anodic aluminum oxides formed in sulfuric acid electrolyte:

*𝐷_𝑖𝑛𝑡_ = 12.1 + 1.99·U for 3 ≤ U ≤ 18* (8)

where *U* is the applied voltage in V. The results of statistical analysis presented in Table 6 demonstrate that a difference between data on the diameter of pores formed during 30 min, interpore distance and pore density in coatings formed in Group 1 and Group 2 processes is statistically significant as $F>F_{0.05}$ and $0.05>P_{0.05}$. A difference in measurements of pore diameters in coatings formed in Group 1 and Group 2 processes during 10 min is not statistically significant. However, a difference between the measurements of pore diameters in all coatings formed during 10 min and 30 min of anodization is statistically significant as $F>F_{0.05}$ and $0.05>P_{0.05}$.

#### 3.1.7. XRD Patterns of Anodized Specimens

**Figure 10:**
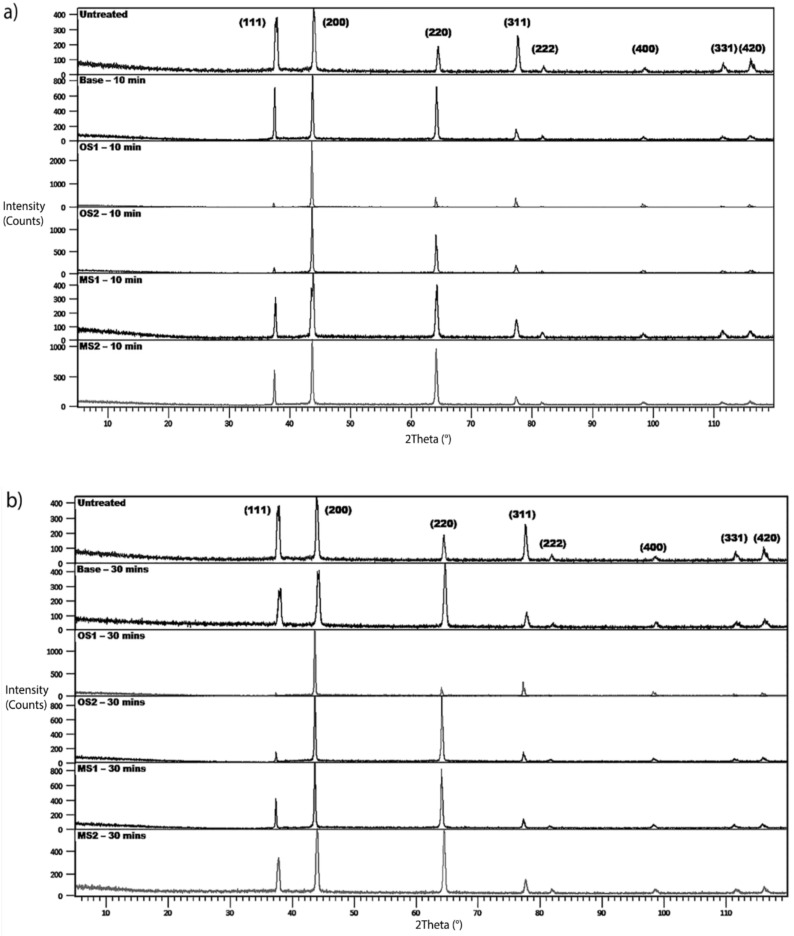
(**a**,**b**). XRD of the untreated specimen alloy and specimens anodized in Base, OS1, OS2, MS1, MS2 process (**a**) for 10 min and (**b**) for 30 min. Measurements collected at an incident angle of 2^0^ using a grazing technique.

XRD of anodic coatings formed in Base, OS1, OS2, MS1, and MS2 processes over 10 min and 30 min are shown in Figure 10. Measurements were conducted at an incident angle of 2° using a grazing technique. For comparison, XRD of untreated specimens are also shown in Figure 10. According to the instrument peak profiles, eight peaks in XRD patterns corresponded to the aluminum face-centered-cubic (fcc) crystal structure. However, shifts and changes in the intensity of diffraction peaks of aluminum oxides were observed in diffraction patterns collected on specimens anodized for 10 min (Figure 10a) and 30 min (Figure 10b). It indicates that amorphous aluminum oxides were formed in the anodizing process.

**Figure 11:**
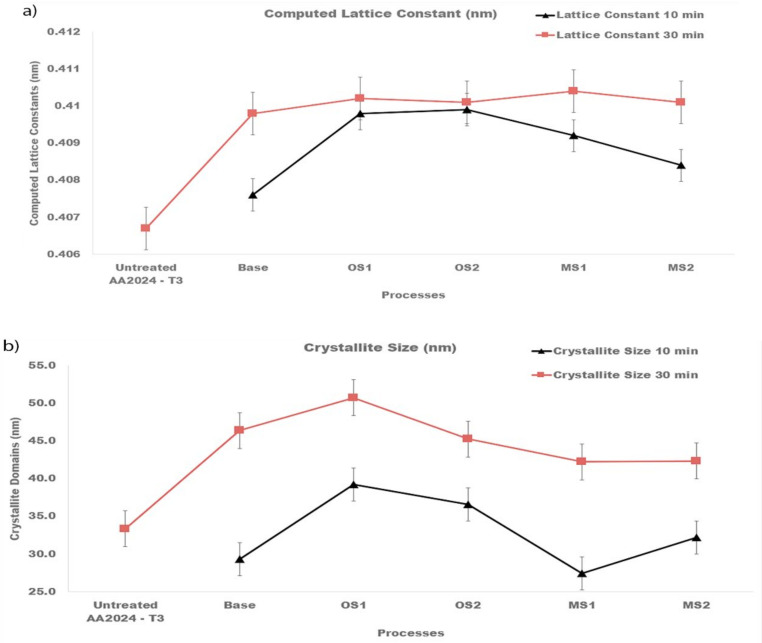
(**a**,**b**). Computed (**a**) lattice constants of the fcc structure of crystallites and (**b**) sizes of crystalline domains for specimens anodized in Base, OS1, OS2, MS1, MS2 process for 10 min and for 30 min. Reported values were averaged over all peaks identified in the diffraction pattern and then averaged over three specimens.

Compared to the untreated specimen, anodization changed the position, width, and intensities of peaks in diffraction patterns. Lattice constants of the fcc structure of anodized specimens are reported in Figure 11a and was computed for both types of XRD measurements from Equation (9) [67]:

(9) $$d_{hkl}=\frac{a}{\surd \left(h^{2}+k^{2}+{\;l}^{2}\right)}$$

where *d_hkl_* is the distance between the adjacent lattice planes in the fcc structure for the peak Bragg angle in nm, a is the lattice constant/parameter in nm, and hkl are the Miller indices for the lattice planes. Results of these calculations presented in Figure 11a were averaged over all fcc peaks in the diffraction pattern and then averaged over three specimens.

Plots in Figure 11a show that anodization increased the lattice constants of crystallites and that they are larger for the entire process. The Scherrer equation, Equation (10) [67], was taken to compute the size of coherently scattering crystalline domains from the peak width:

(10) $$L=\frac{K\lambda }{\beta_{\mathrm{hkl}}\cos\left(\theta_{\mathrm{hkl}}\right)}$$

where L is the mean size of the crystalline domains in nm, K=0.9 is the shape factor, λ = 0.15406 nm is the wavelength of the CuKα radiation, $\theta_{hkl}$ is the peak Bragg angle in radians, $\beta_{hkl}$ is the corrected value of the width at half-maximum (FWHM) of the peak in radians, and hkl are the Miller indices of the crystallographic planes. The measured broadening, Equation (11) [67], of a peak was corrected by the data on the instrumental peak broadening as:

(11) $$\beta_{\mathrm{hkl}}={\left(\beta_{m,\mathrm{hkl}}^{2}-\beta_{\mathrm{ints},\mathrm{hkl}}^{2}\right)}^{1/2}$$

where β_m,hkl_ (rad) is the measured FWHM, and β_inst,hkl_ (rad) is the instrumental FWHM measured using the NIST standard [46]. Results of these calculations shown in Figure 11b were averaged over all peaks identified in the diffraction pattern and then averaged over three specimens. As can be seen from Figure 11b, the size of crystalline domains increased with longer anodization time.

### 3.2. Process Efficiency

Table 7 reports the values of an applied voltage needed to maintain the designed current density in processes Base, OS1, OS2, MS1 and MS2. Differences between the charge transferred per step and the overall charge transferred listed in Table 2 and Table 7 are lying within several percentages as the accuracy of maintaining the constant anodizing current was about 1%. The values of the applied voltage needed to initiate anodization was lower in processes OS1, MS1, and MS2 (Table 7).

**Figure 12:**
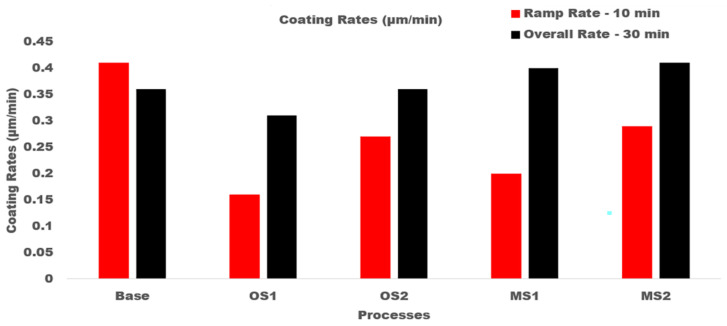
Coating growth rates, µm/min, in processes Base, OS1, OS2, MS1, and MS2. Anodizing times were 10 min and 30 min. Overall growth rates are shown on the right, black column. Multistep processes, MS1 and MS2, provide the highest overall growth rates.

Figure 12 illustrates the coating growth rates, µm/min, computed for 10 and 30 min of anodization for Base, OS1, OS2, MS1, and MS2 processes.

The presented results demonstrate that utilizing multistep ramping of current density at the beginning of anodization allow for a higher growth rate over the 10–30 min period of anodization as well as for the overall growth rate. In particular, the overall growth rates in multistep processes MS1 and MS2 were greater than that of single step ramping processes and greater than that of Base process by 11.3% and 14.5%, respectively. Table 8 presents data on the charge transferred per unit thickness of an anodic coating, C/µm, that were computed for different time intervals as well as over the entire 30 min period of anodization for processes Base, OS1, OS2, MS1, and MS2.

Results presented in Table 8 show that all stepwise processes reduced the overall values of the charge transferred per unit thickness. In particular, it was reduced by 37.5% and 31.3%, respectively, for multistep processes MS1 and MS2. The results of statistical analysis presented in Table 8 demonstrate that a difference between the influence of anodizing processes in Group 1 and Group 2 on the charge per unit thickness is statistically significant as $F>F_{0.05}$ and $0.05>P_{0.05}$.

Table 9 presents anodic coating efficiency, average voltage used, and work required to transfer an electric charge during anodization for processes Base, OS1, OS2, MS1, and MS2. Work was computed using the measurements of voltage presented in Table 7 and anodic process efficiency, η_ox_, was computed using Equation (5).

Results reported in Table 9 demonstrate that stepwise processes reduce the work and applied voltage needed to build an anodic coating and thereby raising the anodization efficiency. Compared to Base, processes MS1 and MS2 are more efficient by 19.7% and 28.2%, respectively. The results of statistical analysis presented in Table 9 demonstrate that a difference between the influence of anodizing processes in Group 1 and Group 2 on the anodic coating efficiency and average voltage is statistically significant as $F>F_{0.05}$ and $0.05>P_{0.05}$. However, a difference in data on the work between processes in Groups 1 and 2 is not statistically significant.

## 4. Conclusions

The presented results demonstrated that starting anodization at a low voltage and then slowly ramping current density increased the growth rate, structure, and service properties of an anodic coating on AA2024—T3 alloy. Five anodizing processes in a sulfuric acid bath were studied: a conventional Base process with a constant applied current density and ramping processes, OS1, OS2, MS1, MS2, applying different magnitudes of current density in either one or five steps. Increasing the number of ramping steps with an incremental rise in current density in processes MS1 and MS2 lowered the oxygen infusion into the coating (Al/O ratio), raised the coating growth rate, reduced the coating porosity, and enhanced the coating abrasion resistance and hardness. Overall, processes MS1 and MS2 were 11.3% and 14.5% faster at producing 1 µm of coating per minute compared to the Base process and formed almost the same thickness in 33% less time. Both multistep ramp processes, MS1 and MS2, produced a thicker coating compared to single-step ramp processes. Multistep processes MS1 and MS2 were, respectively, 19.7% and 28.2% more efficient in building an anodic coating compared to the Base process.

In our previous work [15] we demonstrated that it was possible to improve the properties of anodized AA7075—T6 by gradually increasing the current density during the ramp stage. The results taken together with the presented data on anodization of AA2024—T3 show that benefits of multistep anodization processes are not sensitive to the alloy composition. We therefore expect that the use of multistep anodization with a gradual increase in an applied current density would allow for the development of more efficient anodization processes for other aluminum alloys.

## Figures and Tables

**Table 1:** Composition, wt.% of AA2024—T3 specimens provided by Anacon 1st Choice, 425 W LA Cadena. Riverside, California. The first row is the bulk composition given by the manufacturer. Secondary and Backscattered SEM images at 30,000× were obtained using 10 kV under LED and the height of the specimen stage, WD, of 10.0 mm to observe the surface morphology and measure the local composition with EDS.

Elements (wt.%)	Al	Cu	Mg	Mn	Fe	Zn	Si	O
Manufacturer Data	92.8	4.8	1.4	0.6	0.2	0.1	0.1	-
EDS/Non—Particle Region	80.2 ± 7.4	5.7 ± 1.1	4.4 ± 0.9	1.1 ± 0.7	1.0 ± 0.8	0.7 ± 0.2	0.5 ± 0.1	6.4 ± 3.8
EDS/Particle Region	65.2 ± 6.3	12.3 ± 4.7	6.2 ± 3.2	2.5 ± 1.6	2.4 ± 1.1	0.4 ± 0.1	0.4 ± 0.1	10.6 ± 4.6

**Table 2:** Anodizing processes designed for experiments: a conventional Base process with a constant applied current density and processes with current density applied in one (OS1 and OS2) and five (MS1 and MS2) steps at different magnitudes during the ramp period. The expected values of transferred electric charge computed with the use of Equations (1) and (2).

Process	Mins	Current Density, A/m^2^	Charge, C	Amperage, A
Base	30	180	30,600	17
OS1	10	32	1800	3
	20	180	20,400	17
OS2	10	111	6600	11
	20	180	20,400	17
MS1	2	32	360	3
	2	40	480	4
	2	49	600	5
	2	57	600	5
	2	65	720	6
	20	180	20,400	17
MS2	2	32	360	3
	2	64	720	6
	2	95	1080	9
	2	126	1440	12
	2	158	1800	15
	20	180	20,400	17

**Table 3:** Thickness (µm) of coatings formed in Base, OS1, OS2, MS1, and MS2 processes was measured using an Eddy current meter. Anodic samples for testing were taken during three stages of the process. The samples were taken at 2-min intervals up to and including 10 min, and at the end of the process, 30 min. Statistical analysis of data between processes in Group 1 (Base, OS1, OS2) and Group 2 (MS1, MS2) for the overall process is listed in blue font below.

Thickness (µm)							
Process Time, Min	0 to 2	2 to 4	4 to 6	6 to 8	8 to 10	10 to 30	Overall
Base	1.0 ± 0.3	1.4 ± 0.4	2.0 ± 0.4	3.0 ± 0.5	4.1 ± 0.4	6.8 ± 0.5	10.9 ± 0.7
OS1	0.4 ± 0.2	0.7 ± 0.3	1.1 ± 0.2	1.3 ± 0.3	1.6 ± 0.3	7.7 ± 0.4	9.3 ± 0.5
OS2	0.8 ± 0.3	1.2 ± 0.4	2.0 ± 0.4	2.2 ± 0.4	2.7 ± 0.3	8.0 ± 0.3	10.7 ± 0.7
MS1	0.3 ± 0.1	0.8 ± 0.3	1.5 ± 0.4	1.7 ± 0.4	2.0 ± 0.3	10.2 ± 0.4	12.2 ± 0.4
MS2	0.2 ± 0.1	1.1 ± 0.3	1.5 ± 0.4	2.2 ± 0.3	2.9 ± 0.4	9.7 ± 0.4	12.6 ± 0.5
Groups	Count	Average	P_0_._05_	F	$F_{0.05}$		
Group 1	36	10.3 ± 0.9	2.7 × 10^−14^	100.8	4.2		
Group 2	24	12.4 ± 0.5					

**Table 4:** Testing results of abrasion resistance, microhardness, acid dissolution and weight loss per micron of specimens anodized in Base, OS1, OS2, MS1, MS2 processes. Statistical analysis of data between processes in Group 1 (Base, OS1, OS2) and Group 2 (MS1, MS2) for the overall process is listed in blue font below.

Process	Wt. Loss (mg) in Abrasion Tests	Microhardness (MPa)	Wt. Loss Per Coating Area (mg/dm^2^)	% Loss of Total Coating	Wt. Loss Per Micron (mg/µm)
Base	39.3 ± 4.1	1282.3 ± 57.6	181.0 ± 11.3	99.6	19.3
OS1	34.8 ± 3.8	1147.4 ± 38.3	169.0 ± 4.9	99.2	21.3
OS2	39.2 ± 4.4	1369. 3 ± 20.8	186.0 ± 5.7	99.9	20.2
MS1	26.7 ± 2.0	1398.7 ± 28.1	205.0 ± 8.8	95.2	20.5
MS2	28.7 ± 3.3	1455.1 ± 23.5	227.0 ± 5.2	96.2	21.7
Abrasion					
Groups	Count	Average	P_0.05_	F	$F_{0.05}$
Group 1	18	37.8 ± 4.7	4.3 × 10^−7^	42.8	4.2
Group 2	12	27.7 ± 3.0			
Microhardness					
Groups	Count	Average	P_0.05_	F	$F_{0.05}$
Group 1	18	1266.4 ± 103	2.1 × 10^−5^	26.1	4.2
Group 2	12	1426.9 ± 40			
Acid Dissolution					
Groups	Count	Average	P_0.05_	F	$F_{0.05}$
Group 1	18	178.7 ± 11.0	6.4 × 10^−9^	67.2	4.2
Group 2	12	216.0 ± 13.8			

**Table 5:** The atomic Al/O ratios in coatings formed by anodization over 10 min and 30 min in Base, OS1, OS2, MS1 and MS2 processes. Statistical analysis of data for anodization in Group 1 (Base, OS1, OS2) and Group 2 (MS1, MS2) processes over 10 min and 30 min is listed in blue font below.

Process		Al/O, 10 min		Al/O, 30 min	
Base		0.69 ± 0.04		0.76 ± 0.05	
OS1		0.73 ± 0.05		1.00 ± 0.08	
OS2		0.71 ± 0.05		0.86 ± 0.05	
MS1		0.77 ± 0.06		0.97 ± 0.06	
MS2		0.79 ± 0.06		0.99 ± 0.06	
Al/O Ratio—10 min					
Groups	Count	Average	P_0.05_	F	$F_{0.05}$
Group 1	36	0.71 ± 0.02	2.3 × 10^−5^	21.2	4
Group 2	24	0.77 ± 0.06			
Al/O Ratio—30 min					
Groups	Count	Average	P_0.05_	F	$F_{0.05}$
Group 1	36	0.87 ± 0.12	2.0 × 10^−4^	15.9	4
Group 2	24	0.98 ± 0.06			

**Table 6:** Pore diameter (nm), interpore separation (nm), and pore density (1/µm^2^) in coatings formed by anodization over 10 min and 30 min in Base, OS1, OS2, MS1, and MS2 processes. Values were computed from high-magnification SEM images (100,000×) posted in Figure 3. Computations were conducted with software ImageJ [59]. Statistical analysis of data between processes in Group 1 (Base, OS1, OS2) and Group 2 (M21, MS2) for pore diameter (10 and 30 min), interpore separation and pore density is listed below. Statistical analysis of data between processes in 10 min and 30 min for the pore diameter is also listed in blue font below.

Process	Pore Diameter, nm, 10 min	Pore Diameter, nm, 30 min	Interpore Separation, nm, 30 min	Pore Density, 1/µm^2^, 30 min	
Base	18.3 ± 4.8	33.4 ± 9.2	43.34 ± 0.57	615.2 ± 16.2	
OS1	16.5 ± 4.1	26.7 ± 6.7	23.64 ± 0.70	2067.9 ± 120.4	
OS2	18.4 ± 6.4	29.3 ± 7.2	39.16 ± 0.34	753.1 ± 12.05	
MS1	17.7 ± 5.3	24.9 ± 6.1	22.85 ± 0.85	2214.7 ± 161.6	
MS2	17.9 ± 4.8	34.9 ± 5.6	23.05 ± 0.98	2176.6 ± 179.3	
Pore Diameter—10 min					
Groups	Count	Average	P_0.05_	F	$F_{0.05}$
Group 1	217	18.2 ± 10.5	8.6 × 10^−1^	0.03	3.9
Group 2	160	17.7 ± 9.0			
Pore Diameter—30 min					
Groups	Count	Average	P_0.05_	F	$F_{0.05}$
Group 1	192	28.0 ± 9.6	1.0 × 10^−2^	6.2	3.9
Group 2	120	32.1 ± 8.5			
Pore Diameter—10 min vs. 30 min					
Groups	Count	Average	P_0.05_	F	$F_{0.05}$
10 min	390	17.9 ± 8.9	3.6 × 10^−12^	50.1	3.9
30 min	312	29.6 ± 9.5			
Interpore Distance					
Groups	Count	Average	P_0.05_	F	$F_{0.05}$
Group 1	9	35.4 ± 9. 0	5.0 × 10^−3^	11.1	4.7
Group 2	6	22.9 ± 1.0			
Pore Density					
Groups	Count	Average	P_0.05_	F	$F_{0.05}$
Group 1	9	1146 ± 700	3.0 × 10^−3^	12.8	4.7
Group 2	6	2203 ± 188			

**Table 7:** Data on the applied anodizing current, initial and final voltage, and measurements of charge for each step of Base, OS1, OS2, MS1 and MS2 processes. The presented values were averaged over three runs.

Process	Mins	Amperage, A	Charge, C	Initial Voltage, V	Final Voltage, V
Base	30	17	29,800.0 ± 2332.2	15.7 ± 0.5	16.0 ± 0.2
OS1	10	3	1710.0 ± 98.1	5.8 ± 0.2	9.0 ± 0.3
	20	17	19,755.0 ± 347.6	15.8 ± 0.2	16.3 ± 0.6
OS2	10	11	6005.0 ± 97.9	13.6 ± 0.4	14.6 ± 0.1
	20	17	18,852.0 ± 282.4	15.5 ± 0.2	17.3 ± 0.2
MS1	2	3	333.0 ± 27.3	5.4 ± 0.2	7.4 ± 0.5
	2	4	444.0 ± 25.7	8.6 ± 0.2	8.9 ± 0.6
	2	5	563.0 ± 16.2	9.2 ± 0.2	9.5 ± 0.3
	2	5	598.0 ± 36.8	9.8 ± 0.1	10.2 ± 0.1
	2	6	716.0 ± 94.2	10.5 ± 0.2	10.8 ± 0.1
	20	17	20,165.0 ± 266.9	14.1 ± 0.1	15.9 ± 0.1
MS2	2	3	339.0 ± 16.8	5.5 ± 0.5	7.5 ± 0.7
	2	6	705.0 ± 22.9	9.3 ± 0.5	10 ± 0.6
	2	9	998.0 ± 63.5	11.2 ± 0.4	11.5 ± 0.4
	2	12	1410.0 ± 84.2	13.0 ± 0.7	13.2 ± 0.6
	2	15	1715.0 ± 114.2	13.5 ± 0.7	14.1 ± 0.7
	20	17	19,965.0 ± 201.3	14.4 ± 0.4	16.0 ± 0.4

**Table 8:** Charge transferred per unit thickness of an anodic coating, C/µm. The samples were taken at 2-min intervals up to and including 10 min, and at the end of the process, 30 min for processes Base, OS1, OS2, MS1, and MS2. Statistical analysis of data between processes in Group 1 (Base, OS1, OS2) and Group 2 (M21, MS2) for the overall process is listed in blue font below.

Charge per Unit Thickness (C/µm)							
Process Time, Min	0 to 2	2 to 4	4 to 6	6 to 8	8 to 10	10 to 30	Overall
Base	2040.0	5100.0	3400.0	2040.0	1854.5	3000.0	2733.9
OS1	855.0	1140.0	855.0	1710.0	1140.0	2565.6	2308.1
OS2	1501.3	3002.5	1501.3	6005.0	2402.0	2356.5	2323.1
MS1	1110.7	888.6	804.3	2993.5	2386.7	1977.0	1870.5
MS2	1695.0	783.3	2495.0	2014.3	2450.0	2058.2	1994.6
Groups	Count	Average	P_0.05_	F	$F_{0.05}$		
Group 1	9	2455 ± 227	1.0 × 10^−4^	30.3	4.7		
Group 2	6	1920 ± 74					

**Table 9:** Coating efficiency, η_ox_, of anodization, average voltage, V, and work, kJ, calculated for Base, OS1, OS2, MS1, and MS2 processes. Statistical analysis of data between processes in Group 1 (Base, OS1, OS2) and Group 2 (M21, MS2) for the overall process is listed in blue font below.

Process	Coating Efficiency, (ηox)	Average Voltage, V	Work (kJ)		
Base	0.201 ± 0.006	15.9 ± 0.2	14,334 ± 152		
OS1	0.233 ± 0.005	13.2 ± 0.2	10,475 ± 219		
OS2	0.211 ± 0.001	15.6 ± 0.1	12,926 ± 127		
MS1	0.245 ± 0.011	13.0 ± 0.1	10,862 ± 85		
MS2	0.267 ± 0.003	13.7 ± 0.4	12,039 ± 299		
Efficiency					
Groups	Count	Average	P_0.05_	F	$F_{0.05}$
Group 1	9	0.215 ± 0.015	1.7 × 10^−5^	26.8	4.7
Group 2	6	0.2 56 ± 0.015			
Average Voltage					
Groups	Count	Average	P_0.05_	F	$F_{0.05}$
Group 1	9	14.9 ± 1.3	2.0 × 10^−2^	6.5	4.7
Group 2	6	1 3.3 ± 0.7			
Work					
Groups	Count	Average	P_0.05_	F	$F_{0.05}$
Group 1	9	12,578 ± 1700	1.5 × 10^−1^	2.3	4.7
Group 2	6	11,450 ± 796			

## Data Availability

The data presented in this study are available on request from the corresponding author.
